# Long-term outcomes of blue light laser therapy for epistaxis in patients with hereditary hemorrhagic telangiectasia within a multimodal treatment concept

**DOI:** 10.1007/s00405-025-09919-3

**Published:** 2026-01-20

**Authors:** Christina Heiß, Christopher Bohr, Thomas S. Kühnel, Karolina Müller, Florian Zeman, Kornelia E. C. Andorfer

**Affiliations:** 1 Department of Otorhinolaryngology, University Hospital Regensburg, Franz-Josef-Strauß-Allee 11, 93053 Regensburg, Germany; 2 Center for Clinical Studies, University Hospital Regensburg, Franz-Josef-Strauss-Allee 11, 93053 Regensburg, Germany

**Keywords:** HHT, Hereditary hemorrhagic telangiectasia, Epistaxis, Blue light laser, Laser, Epistaxis Severity Score

## Abstract

**Purpose:**

Hereditary hemorrhagic telangiectasia (HHT) is a genetically inherited vascular disorder transmitted in an autosomal dominant manner. It is defined by the presence of arteriovenous malformations in the nasal mucosa, resulting in severe recurrent epistaxis. This study investigates the long-term efficacy and safety of blue light laser therapy for managing epistaxis in patients with HHT.

**Methods:**

A retrospective analysis was conducted on 71 patients treated between 09/2017 and 02/2023 at the Department of Otorhinolaryngology, University Hospital Regensburg. All patients underwent endonasal treatment using a pulsed diode laser at 445 nm as part of a structured stepwise protocol including nasal care. Of the 86 patients initially treated, 17.4% were excluded due to uncontrollable epistaxis requiring surgery under general anesthesia or concomitant medication.

**Results:**

A total of 351 laser treatments were administered, with no reported occurrences of nasal septal perforation. A modified Epistaxis Severity Score (ESS, range 0–10) decreased by an estimated mean difference of –0.66 (95% CI = –1.05, –0.27). Subgroup analyses revealed that patients with moderate to severe baseline ESS experienced a significant reduction of –1.53 (95% CI = –2.11, –0.95), achieving the minimal important difference (MID) of 0.71. Patients’ satisfaction with their nasal bleeding, measured by the Numeric Rating Scale (0–10), improved significantly from 5.08 to 6.67 (*p* < 0.001).

**Conclusions:**

Our study indicates that blue light laser therapy plus mucosal care is associated with reduced nosebleed severity, improved patient satisfaction and a favorable safety profile, supporting its use as a long-term treatment option for managing HHT in patients with mild to moderate epistaxis.

## Introduction

Hereditary hemorrhagic telangiectasia (HHT), referred to as Rendu-Osler-Weber Syndrome, is a genetically inherited, autosomal dominant vascular disorder. The condition is estimated to occur in 1 out of every 5000 to 8000 individuals globally, classifying it as an orphan disease [1]. HHT is defined by the occurrence of vascular malformations (VMs) and small, dilated blood vessels (telangiectasias), which commonly involve the skin, mucosa and internal organs [2]. Recurrent nosebleeds are both the primary and core symptom of the disease, being observed in virtually all HHT patients by the age of 40 [3]. The severity of epistaxis typically worsens with age, ranging from mild to life-threatening hemorrhages, substantially impairing patients' quality of life [4–6].

Management of epistaxis in HHT relies on two main approaches: regular self-care to maintain humidity and resilience of the mucosa and medical interventions. For patients unresponsive to topical moisturizers, local and systemic intervention including tranexamic acid and bevacizumab are recommended. According to recent guidelines ablative procedures with laser, radiofrequency and sclerotherapy are effective alternatives [6]. Furthermore, early literature has shown first promising results in reducing epistaxis with novel drug therapies.e.g. pazopanib and pomalidomide [7, 8]. At the University Hospital Regensburg epistaxis management in hereditary hemorrhagic telangiectasia follows a structured, multistage protocol. Initial therapy emphasizes mucosal care to preserve the nasal lining, employing non-irritating topical agents such as saline sprays or hyaluronic acid solutions to maintain hydration and prevent crusting [9]. Temporary nasal occlusion (tNO) using hypoallergenic tape improves mucosal moisture and has shown effectiveness in decreasing the frequency and intensity of bleeding, particularly alongside laser therapy [10]. In several specialized HHT centers, laser therapy under local anesthesia is frequently used as a standard approach to treat telangiectasias in the nasal mucosa, with the aim of lengthening recurrence intervals, mitigating bleeding severity [11–13]. In refractory cases, more invasive surgical interventions such as Saunders’ dermoplasty (surgical substitution of the nasal lining with tissue grafts taken from the inner cheek or skin) or Young’s procedure (surgical nasal closure) are considered [6]. This multidisciplinary approach ensures personalized treatment tailored to the severity, recurrence pattern and individual burden of HHT-associated epistaxis.

The TruBlue Laser (Wolf TruBlue™ laser system/ARC Laser GmbH, D-90411 Nuremberg, Germany, wavelength 445 nm) achieves strong absorption in hemoglobin-rich tissues, enabling focused energy delivery to telangiectatic vessels in HHT. This wavelength-dependent interaction may contribute to a more confined thermal effect, minimizing unintended impact on adjacent mucosa. Recent experimental histological data from comparative animal studies indicate that blue light laser application results in a lower penetration depth and reduced lateral spread of thermal damage when compared to conventional bipolar coagulation [14]. Other photoangiolytic lasers currently in use, include the Nd:YAG laser operating at 1064 nm, its frequency-doubled counterpart with a 532 nm wavelength (KTP) and diode lasers, including pulsed variants [9, 15–18].

Nevertheless, to our knowledge, there is no available data regarding long-term effectiveness of blue light laser therapy on epistaxis in patients with Osler disease. Building on the previously discussed considerations, this study focuses on the long-term clinical course and safety of blue light laser therapy for treating recurrent epistaxis in patients with HHT, applied within a structured stepwise treatment concept. Therefore, we retrospectively reviewed the medical records of HHT patients treated with a 445 nm blue light diode laser.

## Materials and methods

### Study design

This retrospective observational study was conducted at the Department of Otorhinolaryngology at the University Hospital Regensburg over approximately 5.5 years (September 2017 to February 2023). During this period, a total of 86 patients diagnosed with HHT (ICD-10 I78.0), were treated using a pulsed diode laser with a wavelength of 445 nm (Wolf TruBlue™ laser system/ARC Laser GmbH, D-90411 Nuremberg, Germany). The clinical application of the TruBlue laser for the treatment of epistaxis in HHT patients has been outlined in a prior study [14]. In routine practice, laser sessions were scheduled individually, either on an on-demand basis according to symptom severity or as prophylactic treatment. This distinction was not systematically documented in the retrospective analysis.

### Inclusion and exclusion criteria

All patients had a confirmed diagnosis of HHT based on the Curaçao criteria (≥ 3 of 4 criteria met). They were included if they had undergone at least two endonasal procedures (OPS-codes 5–985, 5–210.3) using TruBlue laser. Of the 86 patients initially treated, 12.3% (*n* = 10) were excluded from the study due to requiring additional procedures under general anesthesia for uncontrollable epistaxis, indicating a level of disease severity beyond the intended treatment spectrum of this study. Additionally, five patients (*n* = 5, 5.8%) who received either local or intravenous administration of bevacizumab were excluded due to its known effects on reducing epistaxis and to avoid confounding effects on epistaxis outcomes [19–21].

As recommended in current clinical guidelines, all included patients had been receiving standard mucosal care prior to the initiation of laser therapy, as outlined in the introduction. This baseline therapy was not initiated, modified, or advised to be changed at the start or during the course of laser treatment. These measures were applied uniformly as part of baseline management for HHT-related epistaxis [6].

**Fig. 1:**
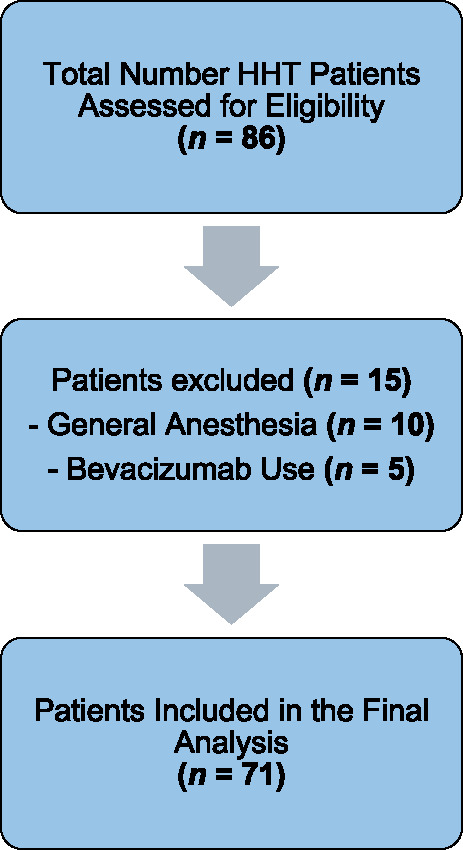
Patient selection flow chart

This resulted in a final study population of 71 patients (Fig. 1), representing the group typically considered suitable for laser therapy in the context of a stepwise treatment approach for mild to moderate epistaxis [16].

### Data collection

Retrospective data were collected from electronic medical records and paper files. Variables included demographic factors (age, sex), number of treatments, and treatment intervals. As this study was observational in nature and utilized routine clinical practice data, no modifications were made to patient treatment protocols, ensuring that standard care remained unchanged. Before focusing exclusively on on blue light laser therapy in this study, other laser techniques may have been used to manage nasal bleeding.

### Outcome measures

To assess the long-term efficacy of blue light laser therapy, several parameters were systematically recorded: modified Epistaxis Severity Score (mESS), Numeric Rating Scale (NRS), hemoglobin (Hb) levels (g/dL), and total energy applied per laser session (J). Hemoglobin levels were measured through conventional laboratory methods. Before each laser treatment, patients were asked to complete both the NRS and ESS questionnaires. The NRS assesses patients’ satisfaction with their nasal bleeding, over the preceding three months, rating on a scale from 0 (extremely dissatisfied) to 10 (completely satisfied). The ESS is a validated, 6-item instrument developed to evaluate the severity of epistaxis in individuals with HHT. It captures patient-reported data on the occurrence, duration, and severity of nosebleeds. Additional items assess the need for medical intervention, the current anemia status and the history of red blood cell transfusions specifically administered due to epistaxis. The ESS was designed to reflect symptoms over the past three months and its minimal important difference (MID) is given in literature with 0,71 [22, 23]. Time intervals between laser treatments were calculated in months. For patients with a follow-up period shorter than three months (*n* = 17, 23.9%), we applied a modified version of the ESS (mESS), interpreting the score relative to the actual interval since the last treatment, in line with a previously described approach [24]. In accordance with established guidelines for HHT patients, ESS can be grouped into severity categories (none: 0–1.00, mild: 1.01–4.00, moderate: 4.01–7.00, severe: 7.01–10.00).

In this study, the first two items of the ESS questionnaire, which assess epistaxis frequency (with response options ranging from less than once per month to several times per day) and duration (with response options ranging from less than 1 min to more than 30 min), were analyzed separately to examine the specific impact of the intervention on these parameters. These assessments provided a comprehensive and retrospective quantitative measure of epistaxis severity, enhancing the clinical decision-making process. ESS responses were analyzed using a dedicated online platform provided by Drexel University College of Medicine (Drexel University, College of Medicine, 2900 W. Queen Lane, Philadelphia, PA 19129, USA; all rights reserved; link: https://apps.med.drexel.edu/HHT-ESS/).

### Statistical analysis

All statistical analyses were conducted using SPSS Statistics Version 28 (IBM Corp., Armonk, NY, USA). Prior to inferential analysis, descriptive statistics were calculated for all relevant variables. General Linear Models (GLM) for repeated measures (Repeated Measures ANOVA) were applied to evaluate changes in mESS, NRS, hemoglobin levels, and total energy applied during each laser session. Each model analyzed within-subject changes over time (comparing the first and last recorded measurements) while accounting for between-subject differences based on treatment frequency (categorized into 1 treatment, 2–5 treatments, and > 5 treatments). Moreover, the GLM for mESS included baseline ESS severity (“none/mild” vs. “moderate/severe”) to evaluate differential treatment effects across severity groups. To control for time-related variability that could impact outcome measures, two covariates were incorporated into the model: (1) the time from baseline to the last measurement, accounting for overall treatment duration, and (2) the time between the last and second-to-last measurements, addressing potential temporal gaps that could bias longitudinal assessments. These covariates ensured that differences in follow-up intervals were appropriately controlled, allowing for a more accurate evaluation of treatment effects over time.

Patients with missing baseline or final values for any outcome variable were excluded from the respective analyses. No data imputation methods were applied. Patients who received only one endonasal treatment were excluded due to the lack of a second measurement for longitudinal comparison. The "1 treatment" group included patients who received two treatments, allowing for a single longitudinal comparison. The "2–5 treatments" group consisted of patients who underwent up to six treatments, providing up to five comparisons, while the " > 5 treatments" group comprised patients with more than six treatments, ensuring more than five longitudinal assessments.

For all models, Estimated Marginal Means including 95% confidence intervals (CI) were calculated. Post-hoc pairwise comparisons were performed using the Least Significant Difference (LSD) method.

In addition to the overall mESS score, the first two sub-items—assessing epistaxis frequency and duration—were analyzed separately to provide a more detailed evaluation of nosebleed characteristics. For this analysis, the values from the initial treatment session and the final documented treatment session were used, reflecting the patients' baseline status and their condition at the end of the treatment course. To assess individual changes over time, the scores from the first and last sessions were directly compared, classifying outcomes as improvement (final score < baseline), worsening (final score > baseline), or no change (final score = baseline). Given the ordinal nature of the data and its non-normal distribution, the Wilcoxon signed-rank test was applied to evaluate paired differences between baseline and final measurements for both sub-items.

## Results

### Demographics

The cohort included 71 patients (60.6% female, 39.4% male). The mean age at study initiation was 46.3 years (SD ± 17.3), with an age range of 8 to 76 years.

### Treatment sessions and timing

A total of 351 laser treatments were administered, with each patient receiving between 2 and 13 treatments. The mean interval between treatments was 5.45 months (range: 0.5–35.7 months).

### Epistaxis severity score

A total of 70 patients were analyzed for mESS. The estimated mean mESS significantly decreased from baseline (3.92, 95% CI: 3.69–4.14) to the final assessment (3.26, 95% CI: 2.89–3.62), with a mean difference of –0.66 (*p* = 0.001), indicating a statistically significant but not clinically relevant reduction in epistaxis severity.

The time interval between the first and last mESS assessments averaged 25 months (SD = 14), with a range of 3 to 55 months.

#### Subgroup analysis

The baseline mESS distribution was as follows: 1.4% of patients were classified as "none," 52.9% as "mild," 44.3% as "moderate," and 1.4% as "severe." For further analysis, these categories were grouped into "none/mild" (*n* = 38, 54.3%) and "moderate/severe" (*n* = 32, 45.7%).

Regarding treatment distribution, 27.1% (*n* = 19) of patients received a single treatment, 45.7% (*n* = 32) received 2–5 treatments, and 27.1% (*n* = 19) received more than five treatments.

In the "moderate/severe" group, mESS decreased from 4.99 (95% CI: 4.65–5.32) at baseline to 3.46 (95% CI: 2.91–4.00) at final assessment, with a mean difference of –1.53 (*p* < 0.001), indicating a clinically meaningful improvement (MID = 0.71). Patients receiving 2–5 treatments also showed a significant mean reduction in mESS of –0.73, from 3.98 (95% CI: 3.66–4.30) to 3.24 (95% CI: 2.72–3.77) (*p* = 0.012). As expected, the "none/mild" group exhibited no statistically significant change in mESS (*p* = 0.426).

**Fig. 2:**
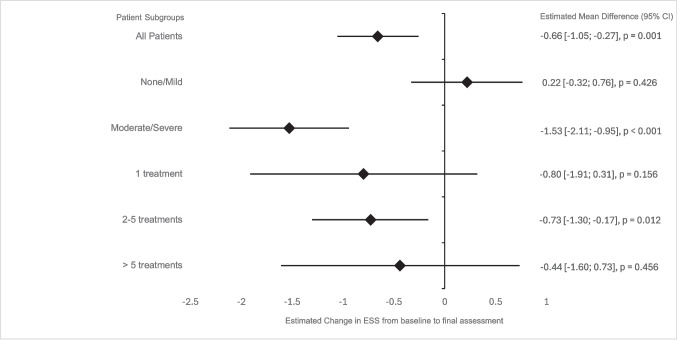
Forest plot illustrating the estimated mean differences in the mESS from baseline to final assessment across patient subgroups, derived from the GLM Mixed ANOVA. Diamonds represent the estimated mean differences, and horizontal lines indicate the 95% confidence intervals (CI). Negative values reflect a reduction in mESS. Statistically significant results are marked with corresponding p-values

The estimated mean differences across patient subgroups, including confidence intervals and significance levels, are visualized in Fig. 2.

### Sub-scores of the modified epistaxis severity score

#### Frequency of nosebleeds

For ESS Question 1, assessing nosebleed frequency (*n* = 67), 46.3% of participants showed an improvement, 19.4% experienced a worsening, and 34.3% showed no change between the initial and final treatment session. The mean score decreased significantly from 4.44 (SD = 1.26) at baseline to 4.00 (SD = 1.52) post-intervention (*p* = 0.017).

#### Duration of nosebleeds

For ESS Question 2, evaluating the duration of nosebleeds (*n* = 66), 27.3% of patients showed an improvement, 15.2% experienced a worsening, and 57.6% showed no change. The mean score decreased from 2.61 (SD = 1.00) at baseline to 2.39 (SD = 0.89) post-intervention without statistical significance (*p* = 0.132).

### Numeric Rating Scale (NRS)

Data from 54 patients were analyzed for NRS changes between the first and last assessments, with subgroup analyses based on the number of treatments received (1 treatment, 2–5 treatments, and > 5 treatments). The estimated mean NRS score increased from 5.08 (95% CI: 4.46–5.69) at baseline to 6.67 (95% CI: 6.05–7.30), resulting in a mean difference of 1.69 (*p* < 0.001). Subgroup analysis showed a significant increase in NRS for patients receiving 2–5 treatments from 5.65 to 7.44 (Δ = 1.78, *p* < 0.001) and for those receiving more than 5 treatments from 4.45 to 6.56 (Δ = 2.11, *p* = 0.047). In contrast, no significant change was observed for patients receiving 1 treatment (Δ = 0.91, *p* = 0.320) (Table 1).

### Hemoglobin levels

Overall, Hb levels (g/dL) remained stable, with no significant difference between baseline (Mea*n* = 12.48, 95% CI: 11.45–13.51) and final measurement (Mea*n* = 12.34, 95% CI: 11.42–13.27) (Δ = –0.14, *p* = 0.771). Subgroup analyses showed consistent Hb levels across treatment groups, with no significant changes observed in any subgroup (see Table 2 for details).

**Table 1:** NRS outcome measures by treatment group

Treatment Subgroups (No.)	Baseline NRS (MEAN*, 95% CI)	Final NRS (MEAN*, 95% CI)	Mean Difference (Final-Baseline)	*p* Value
All patients (*n* = 54)	5.08 (4.46–5.69)	6.67 (6.05–7.30)	1.69 (0.95–2.25)	*p* < 0.001
1 treatment (*n* = 17)	5.12 (3.41–6.83)	6.02 (4.27–7.78)	0.91 (–0.91–2.72)	*p* = 0.320
2–5 treatments (*n* = 21)	5.65 (4.67–6.64)	7.44 (6.43–8.44)	1.78 (0.74–2.82)	*p* < 0.001
> 5 treatments (*n* = 16)	4.45 (2.49–6.41)	6.56 (4.55–8.57)	2.11 (0.03–4.18)	*p* = 0.047

**Table 2:** Hb outcome measures by treatment group (g/dL)

Treatment Subgroups (No.)	Baseline NRS (MEAN*, 95% CI)	Final NRS (MEAN*, 95% CI)	Mean Difference (Final-Baseline)	*p* Value
All patients (*n* = 34)	12.5 (11.45–13.51)	12.3 (11.42–13.27)	–0.14 (–1.08–0.81)	*p* = 0.771
1 treatment (*n* = 8)	13.15 (10.14–16.15)	13.29 (10.60–15.99)	0.15 (–2.60–2.89)	*p* = 0.913
2–5 treatments (*n* = 15)	12.43 (10.95–13.90)	12.47 (11.15–13.80)	0.05 (–1.30–1.39)	*p* = 0.942
> 5 treatments (*n* = 11)	11.86 (9.52–14.20)	11.26 (9.16–13.36)	–0.60 (–2.74–1.54)	*p* = 0.569

### Total energy applied per laser session

The mean total energy per session (measured in Joules) decreased from 196.1 Joules (95% CI: 141.1–251.2) to 107.4 Joules (95% CI: 90.4–124.5) (Δ = –88.7, *p* = 0.003). A significant reduction was observed in patients with > 5 treatments, with energy levels decreasing from 380.5 Joules to 124.3 Joules (Δ = –256.2, *p* = 0.006), while other groups showed no significant change (Table 3).

**Table 3:** Total energy applied per laser session outcome measures by treatment group (J)

Treatment Subgroups (No.)	Baseline NRS (MEAN*, 95% CI)	Final NRS (MEAN*, 95% CI)	Mean Difference (Final-Baseline)	*p* Value
All patients (*n* = 67)	196.1 (141.1–251.2)	107.4 (90.4–124.5)	–88.7 (–145.7 to –31.7)	*p* = 0.003
1 treatment (*n* = 20)	56.7 (−91.4–204.8)	83.0 (37.3–128.8)	26.3 (–127.0–179.6)	*p* = 0.732
2–5 treatments (*n* = 30)	151.2 (71.8–230.6)	114.9 (90.4–139.5)	–36.2 (–118.4–46.0)	*p* = 0.382
> 5 treatments (*n* = 17)	380.5 (205.8–555.2)	124.3 (70.3–178.3)	–256.2 (–437.0 to –75.3)	*p* = 0.006

### Safety outcomes

**Fig. 3:**
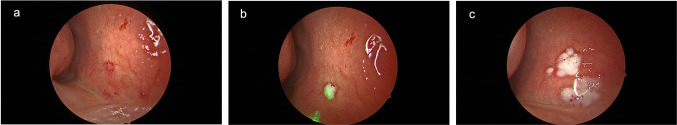
Endonasal treatment of telangiectasias in patients with HHT using the Wolf TruBlue™ laser system (ARC Laser GmbH, D-90411 Nuremberg, Germany). **a** Pre-treatment image showing typical telangiectatic lesions.** b** Intraoperative laser coagulation with precise vessel targeting using the 445 nm TruBlue™ laser. **c** Post-treatment image demonstrating effective coagulation and preservation of surrounding mucosa, minimizing collateral tissue damage

**Fig. 4:**
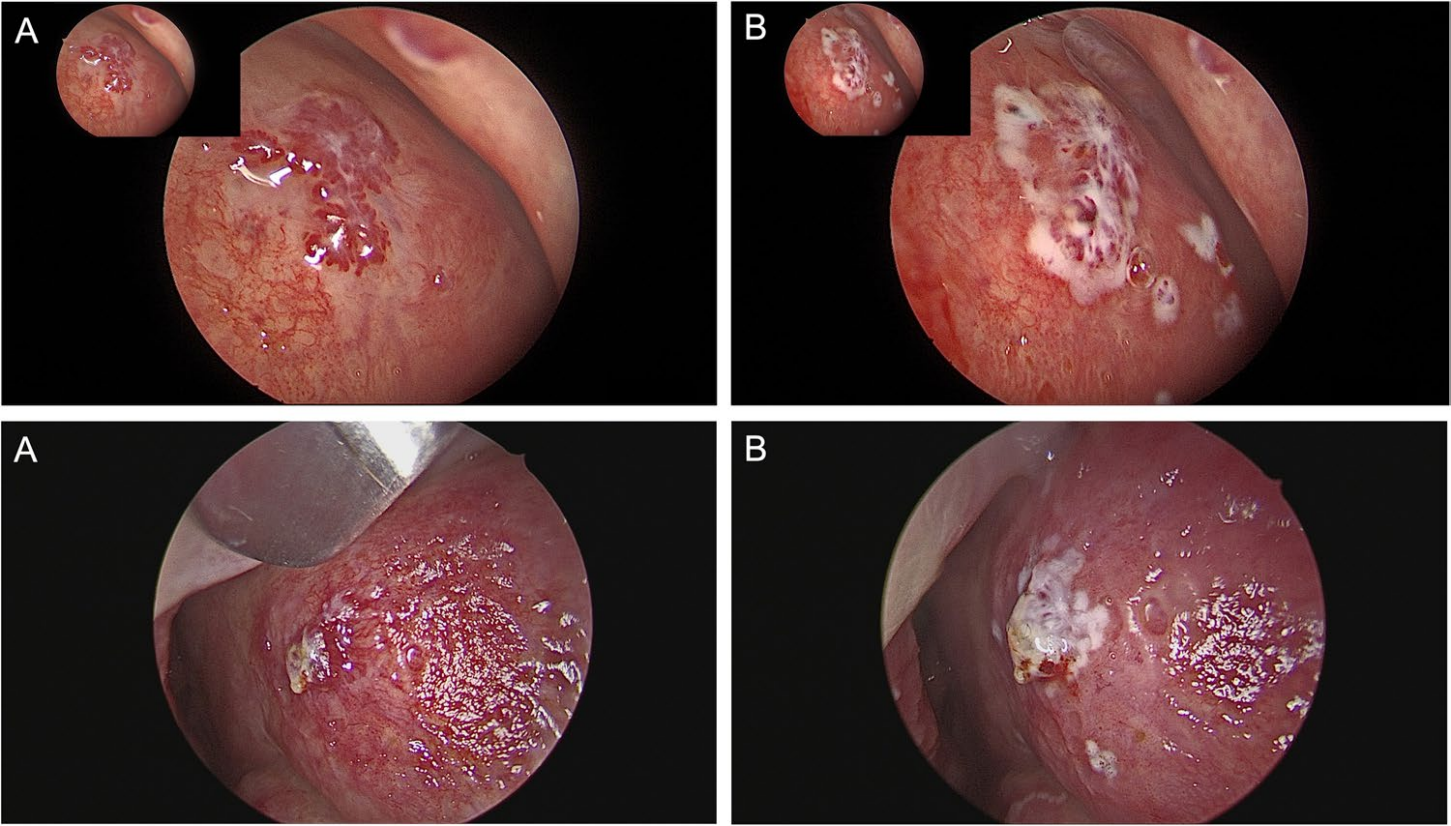
Endonasal visualization of telangiectatic lesions in patients with HHT treated using the Wolf TruBlue™ laser system. **A** Pre-treatment image showing elevated, wart-like telangiectatic lesions. B Post-treatment image illustrating localized lesion coagulation with preserved surrounding mucosa. Treated areas typically appear whitish, without the formation of black coagulation zones. Residual reddish telangiectatic structures may remain visible after treatment due to the laser’s limited penetration depth, consistent with findings for Nd:YAG laser therapy [33]

Among the 71 patients treated, no cases of septal perforation were observed, indicating no adverse events related to septal integrity during the course of treatment. Figure 3 and Fig. 4 illustrate the visual outcomes of blue light laser therapy, showing a typical nasal telangiectasia before and after treatment.

## Discussion

This study investigated the long-term outcomes of blue light laser therapy for managing epistaxis in patients with HHT. Conducted over a period of approximately 5.5 years (September 2017 to February 2023), the study included 71 patients and 351 laser treatments, offering valuable insights into the therapy’s durability, efficacy, and safety. To the best of our knowledge, this represents the longest reported observation time for blue light laser therapy in HHT.

Our findings suggest that blue light laser therapy, alongside consistent standard mucosal care, is associated with stabilization of bleeding patterns and hemoglobin levels over time. This may reflect a relevant therapeutic effect in the context of an often progressive disease like HHT [6]. However, it remains uncertain whether such a progressive course would have been predominant in our study population, as 17.4% of initially treated patients with severe and potentially progressive epistaxis were excluded a priori due to the need for additional interventions, including surgery under general anesthesia or systemic medication. While prior studies primarily focused on short-term outcomes, our results underscore the critical importance of long-term follow-up to fully evaluate the therapy’s benefits and risks [13, 25].

The absence of septal perforations in this cohort supports the favorable safety profile of blue light laser therapy, particularly its ability to minimize thermal damage to surrounding tissues due to its shallow penetration depth (≤ 0.3 mm) and high specificity for hemoglobin. Therefore, blue light laser therapy represents a safe option for treating the typically superficial Osler lesions encountered in patients with HHT. Our findings are consistent with those of Haubner et al., who also described a favorable safety profile for the 445 nm wavelength [14].

Objective outcomes showed modest overall improvements, with the estimated mean mESS decreasing from 3.92 at baseline to 3.26 at final treatment, yielding a mean difference of –0.66. Although this reduction did not meet the MID threshold of 0.71 [22, 23], subgroup analyses revealed significant improvements. Patients with moderate to severe baseline ESS experienced a mean reduction of –1.53 (*p* < 0.001), decreasing from 4.99 (95% CI: 4.65–5.32) to 3.46 (95% CI: 2.91–4.00). Similarly, patients receiving 2–5 treatments achieved the MID, with a mean reduction of –0.73, decreasing from 3.98 (95% CI: 3.66–4.3) to 3.24 (95% CI: 2.72–3.77) (*p* = 0.012). In patients with HHT, stabilizing the frequency and duration of epistaxis over several years is considered a therapeutic success. Since older patients tend to exhibit higher ESS scores due to the progressive worsening of epistaxis with age, maintaining stable ESS values can be rated a significant clinical achievement [3, 6, 26].

A separate analysis of mESS components, focusing on the frequency and duration of nosebleeds, revealed additional insights into the multifaceted nature of epistaxis symptoms. Consistent with the psychometric validation recommendations [27], this approach enhances our understanding of symptom variability. Our results indicate that 46.3% of participants reported improvements in nosebleed frequency, while 27.3% noted improvements in episode duration. Notably, the reduction in nosebleed frequency was statistically significant (*p* = 0.017), whereas changes in duration did not reach significance (*p* = 0.132). This discrepancy may reflect distinct treatment effects. While laser therapy appears to reduce the number of vulnerable vascular lesions and thereby lowers epistaxis frequency, it may exert limited influence on factors determining the duration of individual episodes. However, the retrospective design and reliance on self-reported ESS data present limitations, including potential recall bias and a sample size that may not detect smaller yet clinically meaningful changes in nosebleed duration.

In addition to improvements in mESS, patients treated with blue light laser therapy maintained stable hemoglobin levels throughout the treatment period. While causality cannot be inferred due to the lack of a control group and the heterogeneity of HHT progression, this finding remains clinically meaningful. Chronic anemia is a characteristic complication of HHT, typically caused by recurrent nasal or gastrointestinal bleeding. Without effective treatment, hemoglobin levels tend to decline over time [6, 28]. Thus, hemoglobin stability may indicate a therapeutic benefit and contribute to improved quality of life, as supported by the significant increase in Numeric Rating Scale scores observed in this study, rising from 5.08 to 6.67 (*p* < 0.001, Δ = 1.69). These findings align with previous research, including Merlo et al. (2014), which highlighted a direct correlation between the severity of epistaxis and reduced health-related quality of life (HR-QoL) [29].

A significant reduction in total energy delivered per laser session was also observed, decreasing from 196 Joules (95% CI: 141–251) at the first session to 107.4 Joules (95% CI: 90–125) at the final session (Δ = –88.70, *p* = 0.003). In patients undergoing more than five treatments, energy application declined even further, from 380.5 Joules (95% CI: 205.8–555.2) to 124.3 Joules (95% CI: 70.3–178.3) (Δ = –256.20, *p* = 0.006). This trend may reflect a progressively more efficient and tissue-conserving treatment approach. Possible explanations include reduced lesion activity or changes in vascular morphology; however, longitudinal lesion behavior could not be assessed due to the absence of standardized monitoring methods. Since clinical severity in HHT correlates more strongly with the frequency and intensity of bleeding than with lesion count, epistaxis severity was chosen as the primary outcome measure in this study.

A total of 17.4% of initially treated patients (15 out of 86) were excluded due to the need for additional invasive interventions or concurrent bevacizumab therapy, indicating that laser therapy alone may be insufficient in more severe cases of HHT-related epistaxis. This finding supports a previously published stepwise treatment algorithm, which recommends escalating interventions based on disease severity [16]. According to this concept, laser therapy is primarily suitable for patients with mild to moderate epistaxis, while more advanced cases, particularly those with high-flow arteriovenous shunts or acute bleeding, may require bipolar radiofrequency coagulation [13, 16, 25]. The final study population, therefore, reflects patients with milder disease severity, in whom laser treatment is typically considered appropriate. We acknowledge that this selection may limit generalizability and introduce bias toward more favorable natural courses.

In line with current clinical guidelines, all patients received standard mucosal care as part of baseline epistaxis management [6]. While these supportive measures likely contributed to symptom control, their individual effect cannot be isolated within a retrospective design.

Certain limitations must be acknowledged. The retrospective design introduces biases, including recall bias and dependence on the mESS. While the ESS is widely used in HHT research, it does not fully capture the variability and severity of epistaxis or its short-term fluctuations, and it has been formally validated only in the English language, which may limit its psychometric validity in non-English-speaking populations. Future prospective studies should consider the implementation of validated tools, such as the recently developed Nasal Outcome Score for Epistaxis in HHT (NOSE HHT) and implement real-time symptom tracking to improve assessment accuracy, providing a more comprehensive assessment of clinical severity and patient-reported quality of life impacts [30]. Additionally, the inclusion of both on-demand and prophylactic treatments reflects routine clinical practice but resulted in a heterogeneous cohort. Due to the retrospective design, subgroup-specific analyses were not feasible and should be addressed in future prospective studies with standardized documentation of treatment intent and timing. In conclusion, the relatively small, single-center sample of 71 patients may limit the generalizability of these findings to the broader HHT population. Larger, multicenter studies are needed to confirm these results and to better capture variability across different patient subgroups.

Although no septal perforations were observed, other potential adverse effects, such as procedural discomfort or mucosal healing complications, were not systematically documented. This highlights the need for more comprehensive reporting of side effects in future studies. In particular, crusting is a known complication of thermic nasal interventions and may affect mucosal healing and patient comfort [31]. Its relevance in the context of blue light laser therapy has yet to be systematically investigated. Furthermore, the long-term effects of repeated laser interventions on mucosal integrity remain unclear. Histological studies of treated tissues could provide valuable insights into cellular alterations and help optimize laser parameters for long-term outcomes. Future research should also aim to directly compare the tissue effects of different laser systems using standardized histopathological assessments, in order to evaluate relative mucosal preservation and thermal damage.

Taken together, the present findings describe clinical outcomes in a selected group of patients with mild to moderate HHT-related epistaxis managed with blue light laser therapy as part of a multimodal treatment strategy. While the retrospective design and absence of a control group limit causal interpretation, the observed trends suggest that blue light laser therapy may contribute to symptom stabilization in mild to moderate cases. Instruments such as the Osler Calendar may facilitate structured symptom tracking and improve patient-provider communication [32].

## Conclusion

This retrospective study suggests that blue light laser therapy, as part of a structured stepwise protocol with standardized nasal care, may contribute to reduced epistaxis severity and stabilization of hemoglobin levels in patients with mild to moderate HHT. Subgroup analyses revealed that patients with higher baseline severity showed the most pronounced symptom improvements, indicating that selected individuals with more severe presentations may still benefit from localized blue light laser treatment. Observed trends over a 5.5-year study period support a favorable safety profile, with no cases of septal perforation, potentially reflecting the laser’s shallow penetration depth and selective absorption by hemoglobin. Prospective, multicenter trials with standardized protocols, systematic adverse event reporting, and histological assessments will be essential to evaluate long-term mucosal effects and to refine treatment parameters.

## Data Availability

The datasets used and analyzed during the current study are available from the corresponding author upon reasonable request.

## Abbreviations

HHT: Hereditary Hemorrhagic Telangiectasia
ESS: Epistaxis Severity Score
mESS: Modified version of the ESS
NRS: Numeric Rating Scale
Hb: Hemoglobin
MID: Minimal important difference
VMs: Vascular malformations
